# Platelet Apoptosis in Adult Immune Thrombocytopenia: Insights into the Mechanism of Damage Triggered by Auto-Antibodies

**DOI:** 10.1371/journal.pone.0160563

**Published:** 2016-08-05

**Authors:** Nora P. Goette, Ana C. Glembotsky, Paola R. Lev, Matías Grodzielski, Geraldine Contrufo, Marta S. Pierdominici, Yesica R. Espasandin, Dardo Riveros, Alejandro J. García, Felisa C. Molinas, Paula G. Heller, Rosana F. Marta

**Affiliations:** 1 Departamento de Hematología Investigación. Instituto de Investigaciones Médicas A. Lanari-IDIM, Universidad de Buenos Aires (UBA), Consejo Nacional de Investigaciones Cientificas y Tecnicas (CONICET), Buenos Aires, Argentina; 2 Departamento de Hematología, Hospital Ramos Mejía, Buenos Aires, Argentina; 3 Departamento de Hematología, Centro de Educación Médica e Investigaciones Clínicas “Norberto Quirno” (CEMIC), Buenos Aires, Argentina; 4 Laboratorio de Citometría de Flujo, Centro de Educación Médica e Investigaciones Clínicas “Norberto Quirno” (CEMIC), Buenos Aires, Argentina; JAPAN

## Abstract

Mechanisms leading to decreased platelet count in immune thrombocytopenia (ITP) are heterogeneous. This study describes increased platelet apoptosis involving loss of mitochondrial membrane potential (ΔΨm), caspase 3 activation (aCasp3) and phosphatidylserine (PS) externalization in a cohort of adult ITP patients. Apoptosis was not related to platelet activation, as PAC-1 binding, P-selectin exposure and GPIb-IX internalization were not increased. Besides, ITP platelets were more sensitive to apoptotic stimulus in terms of aCasp3. Incubation of normal platelets with ITP plasma induced loss of ΔΨm, while PS exposure and aCasp3 remained unaltered. The increase in PS exposure observed in ITP platelets could be reproduced in normal platelets incubated with ITP plasma by adding normal CD3^+^ lymphocytes to the system as effector cells. Addition of leupeptin -a cathepsin B inhibitor- to this system protected platelets from apoptosis. Increased PS exposure was also observed when normal platelets and CD3^+^ lymphocytes were incubated with purified IgG from ITP patients and was absent when ITP plasma was depleted of auto-antibodies, pointing to the latter as responsible for platelet damage. Apoptosis was present in platelets from all patients carrying anti-GPIIb-IIIa and anti-GPIb auto-antibodies but was absent in the patient with anti-GPIa-IIa auto-antibodies. Platelet damage inversely correlated with platelet count and decreased during treatment with a thrombopoietin receptor agonist. These results point to a key role for auto-antibodies in platelet apoptosis and suggest that antibody-dependent cell cytotoxicity is the mechanism underlying this phenomenon.

## Introduction

Immune thrombocytopenia (ITP) is an autoimmune condition in which defects in immune self-tolerance lead to humoral and cellular abnormal responses comprising auto-antibody production and cytotoxic effects [1]. These abnormal immunological patterns are responsible for increased platelet destruction as well as decreased megakaryopoiesis and thrombopoiesis, [2, 3] both leading to a thrombocytopenic state.

A large number of studies established the crucial role of auto-antibodies in ITP pathogenesis, demonstrating that main platelet antigenic targets are the fibrinogen receptor glycoprotein complex (GP) IIb-IIIa and the von Willebrand receptor GPIb-IX [4, 5] while a lower proportion of auto-antibodies react with the collagen receptors GPIa-IIa and GPIV [6]. Antibody-bound-platelet phagocytosis by the reticuloendothelial system is the primary pathogenic mechanism by which auto-antibodies induce thrombocytopenia, although lysis mediated by complement activation on antibody-bound platelets seems to have also a role in ITP [7, 8]. Beyond auto-antibodies, other mechanism involving direct T-cell mediated cytotoxicity was also shown to participate in platelet destruction [9, 10].

Similarly to nucleated cells, platelet life span is controlled by an intrinsic apoptotic program, being major players in this process the anti-apoptotic protein BcL-xL and pro-apoptotic proteins Bak and Bax [11]. Pro- and anti-apoptotic protein unbalance triggers mitochondrial outer membrane permeabilization (MOMP) that is followed by mitochondrial inner membrane potential collapse (ΔΨm), efflux of cytochrome c into the cytoplasm, activation of caspase 3 and 9, phosphatidylserine (PS) externalization and microparticle shedding [12]. Since some of these events also take place during platelet activation, markers of platelet apoptosis should be carefully analyzed.

Previous studies have assessed the contribution of platelet apoptosis to ITP pathogenesis. Platelet apoptosis was first demonstrated in an animal model of ITP, in which injection of anti-GPIIb antibodies triggered features of platelet apoptosis, including ΔΨm, PS exposure and caspase activation, in murine platelets [13]. Concerning human ITP, evidence of platelet apoptosis, including caspase 3, 8 and 9 activation, was shown in children with acute ITP, which was ameliorated by intravenous immunoglobulin infusion [14], whereas another study revealed that platelets from adult chronic ITP patients displayed increased phosphatidylserine exposure associated with dendritic cell dysfunction, although other markers of platelet apoptosis could not be demonstrated in this cohort [15].

The aim of the present work was to deepen into the study of platelet apoptosis in adult ITP patients, to evaluate its relationship with clinical and biochemical parameters including the presence and type of auto-antibody, and to investigate possible triggering mechanisms. Our results provide new evidence involving auto-antibodies as main contributors to platelet apoptosis in ITP.

## Materials and Methods

### Patients and blood samples

Twenty-four patients with chronic ITP (median age, 42 years, range 21–80) diagnosed according to current criteria [16] were included. This project was approved by the Ethics Committee from Instituto de Investigaciones Médicas “Alfredo Lanari” on May 3, 2010. Clinical and laboratory data are presented in Table 1.

**Table 1:** Clinical and laboratory data from ITP patients.

Patient	Platelets	Auto-antibody directed to:			BS	Treatment
N°	(x 10^9^/L)	GPIIbIIIa	GPIbIX	GPIaIIa		
1	38	-	-	-	2	None
2	30	-	-	-	1	Corticosteroids
3	52	-	-	+	2	None / Spl.
4	85	-	-	-	0	Danazol
5	46	-	-	-	2	None / Spl.
6	6	+	-	-	2	None
7	30	-	-	-	1	Corticosteroids
8	16	-	+	-	2	Corticosteroids/Spl.
9	29	-	-	-	2	Corticosteroids/Spl
10	50	+	-	-	1	Corticosteroids
11	30	-	-	-	1	None
12	22	-	-	-	2	Cortic/IVIg/Spl
13	63	-	-	-	2	Corticosteroids
14	8	-	-	-	2	None / Spl.
15	65	-	-	-	0	Corticosteroids
16	22	-	-	-	2	Corticosteroids
17	20	-	-	-	0	None
18	62	-	-	-	2	Corticosteroids
19	50	+	-	-	1	None
20	53	+	-	+	1	Corticosteroids
21	37	-	-	-	2	Corticosteroids
22	50	ND	ND	ND	1	None
23	23	ND	ND	ND	1	None
24	40	+	-	-	2	Azathioprine/Spl.

Platelet count and treatment at the time of study. BS: Bleeding scale; Spl: splenectomy; Cortic: Corticosteroids; IVIg: intravenous immunoglobulin; ND: not done.

Bleeding score was evaluated according to the ITP Bleeding Scale (IBLS) proposed by Page and col [17]. Briefly, grade 0 corresponded to no bleeding manifestations, grade 1, to mild bleeding symptoms assessed in at least one of nine anatomical sites including skin, mucosa, gastrointestinal, urinary and gynaecological tract, lungs and central nervous system, and grade 2 corresponded to severe bleeding in at least one of the mentioned anatomical sites.

Blood samples were obtained after written informed consent in accordance with the Declaration of Helsinki as follows: 5 mL were collected into ACD-EDTA (citric acid 71.4 mmol/L, sodium citrate 85 mmol/L, dextrose 11.1 mmol/L, EDTA 5 mmol/L) for apoptosis studies, 5 mL into 3.8% sodium citrate for activation studies and preparation of recalcified plasma and 2.5 mL into 342 mmol/L EDTA for cell count.

### Sample preparation

- Fresh human platelets obtained by differential centrifugation either from ITP patients or normal volunteers, were washed in CGS Buffer (130 mmol/L ClNa, 12.9 mmol/L sodium citrate, 30 mmol/L glucose, pH 6.5), and resuspended in Binding Buffer (0.01 mol/L Hepes/NaOH, pH 7.4, 0.14 mol/L NaCl, 2.5 mmol/L CaCl_2_) to a density of 10^7^ platelets/mL.
- Preparation of recalcified plasma, IgG purification and adsorption of auto-antibodies from ITP plasma were conducted as described previously [3]. Complete depletion of auto-antibodies from platelet-adsorbed (PA) plasma was confirmed by ELISA measurement, as detailed below. Normal control plasmas were processed simultaneously under the same conditions.
- Apoptosis was evaluated in ITP platelets either unstimulated or with the addition of calcium ionophore (A23187) (SIGMA, St. Louis, MO) for 3 minutes at room temperature. A23187 final concentrations were 1 to 3 μmol/L, except for active caspase 3 assays, where 6 to 10 μmol/L A23187 were also tested.
- For normal platelet apoptosis studies in the presence of ITP plasma, normal platelets were resuspended in recalcified either normal or ITP plasma to a final concentration of 10^7^ platelets/mL and incubated at room temperature during 1 hour. Platelets were then washed, resuspended in binding buffer and evaluated for apoptosis markers.
- Interaction between normal platelets and autologous normal CD3^+^ lymphocytes in the presence of ITP plasma, purified IgG or PA-plasma: normal CD3^+^ lymphocytes were isolated by mononuclear cell density gradient centrifugation and subsequent immunomagnetic positive selection (Miltenyi-Biotec, Bergisch Gladbach, Germany). Cell purity assessed by flow cytometry analysis of fluorescein isothiocyanate (FITC)-conjugated CD3 and phycoerythrin (PE)-conjugated CD45 (both from BD Biosciences, San Diego, CA, USA) was over 98%. Then, 5x10^5^ CD3^+^ lymphocytes were incubated with 5x10^6^ autologous platelets in 100 μL of either ITP or control recalcified plasma, as well as its platelet-adsorbed fraction and purified IgG fraction, during 1 hour at 37°C. Cells (CD3^+^ lymphocytes and platelets) were washed, resuspended in binding buffer and tested for apoptotic parameters. To analyze the role of antibody dependent cell-mediated cytotoxicity (ADCC) in platelet damage in this system, 20 μmol/L leupeptin (SIGMA, St. Louis, MO) was also added to inhibit cathepsin B [18–19] that could be released by CD3^+^ lymphocytes. Results represent the average of at least two independent experiments.

### Apoptosis studies

Phosphatidylserine exposure on the platelet surface: samples were incubated for 15 minutes with 5 μL (FITC)-conjugated Anexin V (BD Biosciences) and 3 μL PE-conjugated anti-CD41a (BD Biosciences Pharmingen, San Diego, CA, USA) and acquired within 1h on a FACSCanto II flow cytometer (BD Biosciences). Results were expressed as percentage of positivity.

Mitochondrial electrochemical potential (ΔΨm): loss of ΔΨm was determined using the cell penetrating lipophilic cationic fluorochrome JC-1 (Molecular Probes, Eugene, OR, USA). Platelets were incubated for 20 minutes with 10 μg/mL JC-1 diluted with PBS supplemented with 1% BSA and immediately analyzed by flow cytometry. Results were expressed as percentage of events with low red fluorescence, reflecting the decrease in the content of JC-1 aggregates when the inner mitochondrial membrane becomes depolarized.

Active caspase 3: caspase 3 activation was measured by the carboxyfluorescein-labeledfluoromethyl ketone tetrapeptide inhibitor of caspase 3 (FAM-DEVD-FMK; Chemicon International, Temecula, CA, USA) that specifically and covalently binds to active caspase 3. Briefly, 10^5^ platelets were incubated in the dark for 1 hour at 370°C with 1:150 diluted FAM-DEVD-FMK solution. After washing, platelets were analyzed by flow cytometry. Anti-CD42b-PE (clone HIP1) (BD Biosciences Pharmingen) was used to identify platelet population. Results were expressed as percentage of platelets with active caspase 3 (aCasp3).

Apoptotic parameters in ITP platelets were considered altered when exceeded the mean+2SD of control samples. Platelet apoptotic status was defined abnormal when at least two of the three markers were above the normal limit.

### Platelet activation studies

PAC-1 binding and P-selectin expression were evaluated as described elsewhere [20] either in resting conditions or after stimulation with 20 μmol/L ADP or 20 μmol/L thrombin receptor activator peptide (TRAP). For GPIb-IX internalization assay, platelets were fixed with 1% PFA for 15 minutes. After a washing step, GPIb-IX on the cell surface was identified by incubation with anti-CD42b-PE and flow cytometry analysis. GPIb-IX internalization was calculated as percent of decrease in mean fluorescence intensity (MFI) of activated (A) compared to resting (R) platelets.

### Auto-antibody evaluation

Specificity of auto-antibodies was evaluated on EDTA-anticoagulated plasma and, in some cases, in platelet-adsorbed plasma, processed using PAKAUTO kit (GTI Diagnostics Inc., Waukesha, WI, USA), according to the manufacturer´s instructions. This methodology allows the detection of auto-antibodies (IgG, IgM or IgA subclasses), identifying those directed against GPIIb-IIIa, GPIb-IX and GPIa-IIa.

### Reticulated platelets

The immature platelet fraction was determined by flow cytometry using thiazole orange (TO, Sigma-Aldrich, St Louis, MO, USA). Briefly, platelets were incubated with TO, 10 ng/mL for 1 hour at RT in the dark. Platelets were identified using PE-conjugated CD41.

### Statistical analysis

Data are presented as median and range. Variables were analyzed for normality and equality of variances using Shapiro-Wilks and F-test, respectively. Differences between data from ITP samples and normal controls were assessed using unpaired t-test or Mann–Whitney test. When indicated, groups were compared in a paired way using paired t-test or Wilcoxon signed rank test. Chi-square or Fisher’s exact test were used to analyze categorical variables. Relationship between two sets of variables was determined by Pearson or Spearman correlation. P values less than 0.05 were considered statistically significant.

## Results

### Platelets apoptosis is increased ITP patients

**Fig 1:**
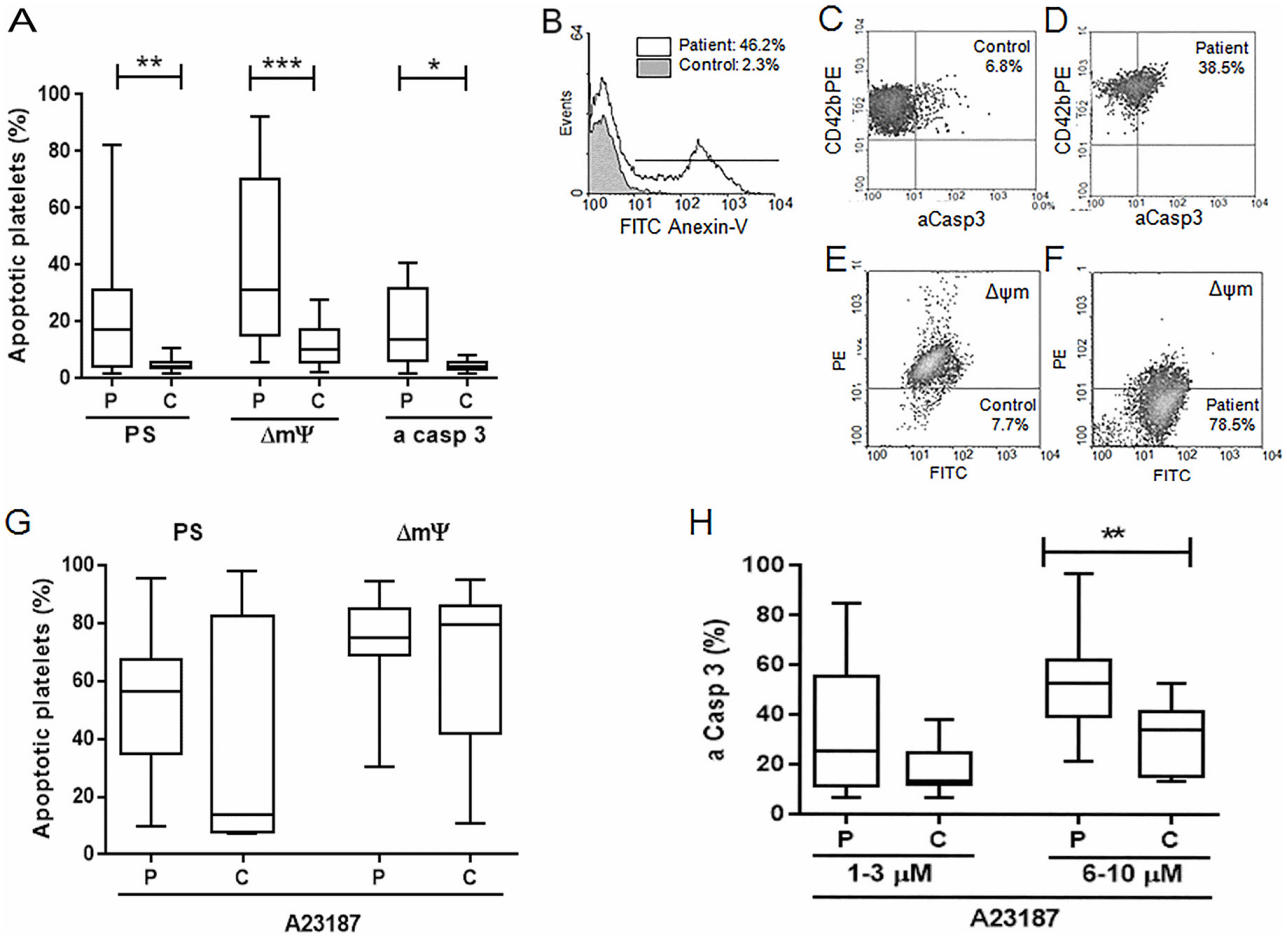
Apoptosis markers in ITP platelets. A) Unstimulated platelets from ITP patients were washed and incubated either with FITC-anexin-V to detect phosphatidylserine (PS) exposure (n = 21), JC-1 to evaluate loss of ΔΨm (n = 24) or FAM-DEVD-FMK to measure active caspase 3 (aCasp3) (n = 12) as described in Materials and methods. Samples were analyzed by flow cytometry within 1 hour of processed. Box plot represent percentage of platelets displaying apoptotic markers in ITP patients (P) and controls (C). Wilcoxon signed rank test *p<0.05, **p<0.01, ***p<0.001. Representative examples of FITC-anexin-V binding (B), active caspase 3 detection (C, D) and loss of ΔΨm measurement (E, F) in control and ITP platelets are shown. G) Platelet apoptosis was induced by the addition of 1–3 μmol/L A23187 and evaluated by PS exposure (p = NS, n = 20) and ΔΨm measurement (p = NS, n = 23). H) Active caspase 3 was evaluated after apoptosis induction with 1–3 and 6–10 μmol/L A23187 (p = NS and **p<0.01, respectively).

To determine whether platelet apoptosis occurs in our cohort of adult patients with ITP, we evaluated PS exposure, ΔΨm, and levels of active caspase 3 in ITP platelets in resting conditions. Platelets from ITP patients displayed increased PS expression, loss of ΔΨm and higher aCasp3 (Fig 1A). Examples of the apoptotic parameters evaluated in normal and ITP platelets are shown in Fig 1B–1F.

### ITP platelets show increased levels of active caspase 3 after calcium ionophore stimulation

After stimulation with A23187, a known inducer of apoptosis [21], ITP platelets showed similar levels of PS expression and ΔΨm than normal controls (n = 20 and n = 23, respectively, both p = NS) (Fig 1G). However, higher sensitivity to the apoptotic stimulus was evidenced by increased levels of aCasp3 which reached statistical significance at high calcium ionophore concentrations (n = 12, Fig 1H).

### Markers of platelet activation in ITP

**Fig 2:**
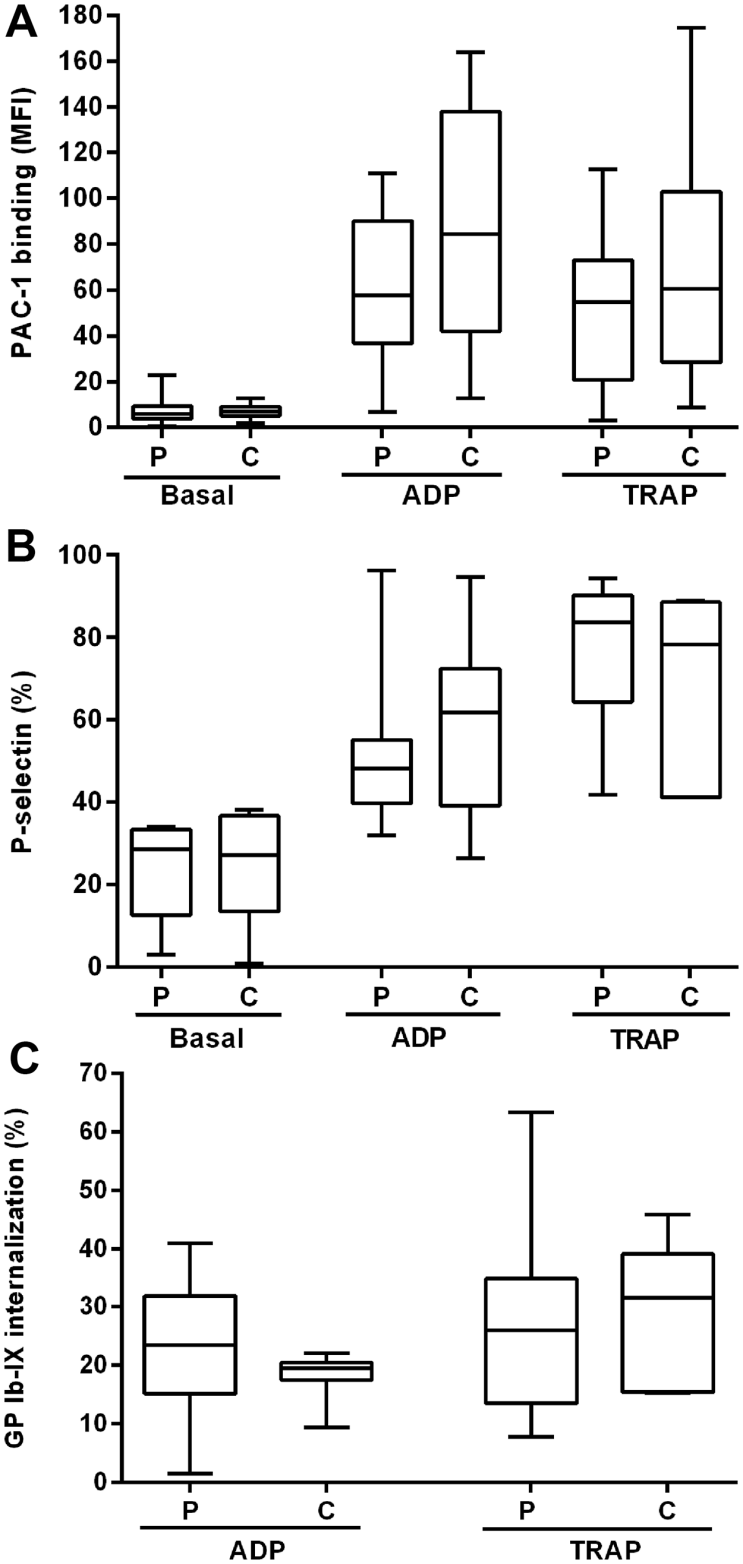
Basal and agonist-induced platelet activation in ITP patients. Unstimulated platelets and platelets activated with 20 μM ADP or TRAP were evaluated for (A) GPIIb-IIIa activation by incubation with FITC-PAC-1 (results are expressed as MFI) and (B) percentage of P-selectin externalization by incubation with FITC-CD62P. C) Percentage of GPIb-IX internalization was calculated by measuring GPIb-IX expression before and after agonist-induced platelet activation. None of these parameters were significantly altered in ITP platelets (P) compared to control platelets (C) (Mann-Whitney test).

Basal levels of platelet activation did not differ between chronic ITP patients and healthy controls when evaluated by PAC-1 binding to GPIIb-IIIa (n = 16). After stimulation with ADP or TRAP, PAC-1 binding to ITP platelets tended to be lower than normal controls although differences were not statistically significant (n = 16, Fig 2A). ITP platelets did not show increased expression of P-selectin either in resting conditions or when stimulated with ADP or TRAP (n = 8, Fig 2B). Moreover, internalization of GPIb-IX after ADP and TRAP stimulation was similar between patients and controls (n = 9 for both agonists, Fig 2C).

### Effect of ITP plasma on apoptosis of normal platelets

**Fig 3:**
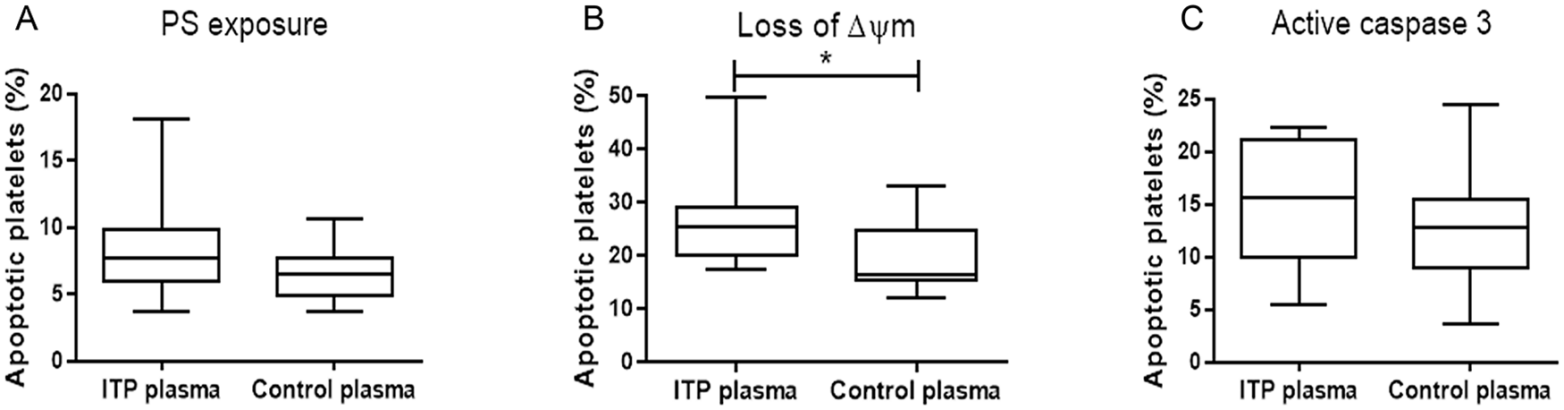
Apoptosis markers in normal platelets incubated with ITP plasma. Unstimulated normal platelets were washed and incubated either with ITP or control plasma during 1 hour. Then, platelets were washed and incubated with FITC-anexin-V, JC-1 or FAM-DEVD-FMK as described in Materials and Methods, and analysed by flow cytometry within 1hour of processed. Box plot represent percentage of apoptotic platelets measured as A) PS exposure (Mann-Whitney test p = NS, n = 10), B) loss of ΔΨm (*p<0.05, n = 12) and C) aCasp3, (p = NS, n = 18).

In order to test whether a plasmatic factor such as auto-antibodies present in ITP plasma samples could induce platelet apoptosis by itself, we tested apoptosis parameters in normal platelets after 1 hour incubation with either ITP or normal plasma. A trend towards higher apoptosis was observed in normal platelets incubated with ITP plasma compared to those incubated with normal plasma. This increase was statistically significant only for ΔΨm (n = 18, Fig 3A–3C), which, in our hands represents a more sensitive apoptotic parameter.

### Effect of ITP plasma on normal platelet phosphatydilserine expression in the presence of autologous normal CD3^+^ lymphocytes

**Fig 4:**
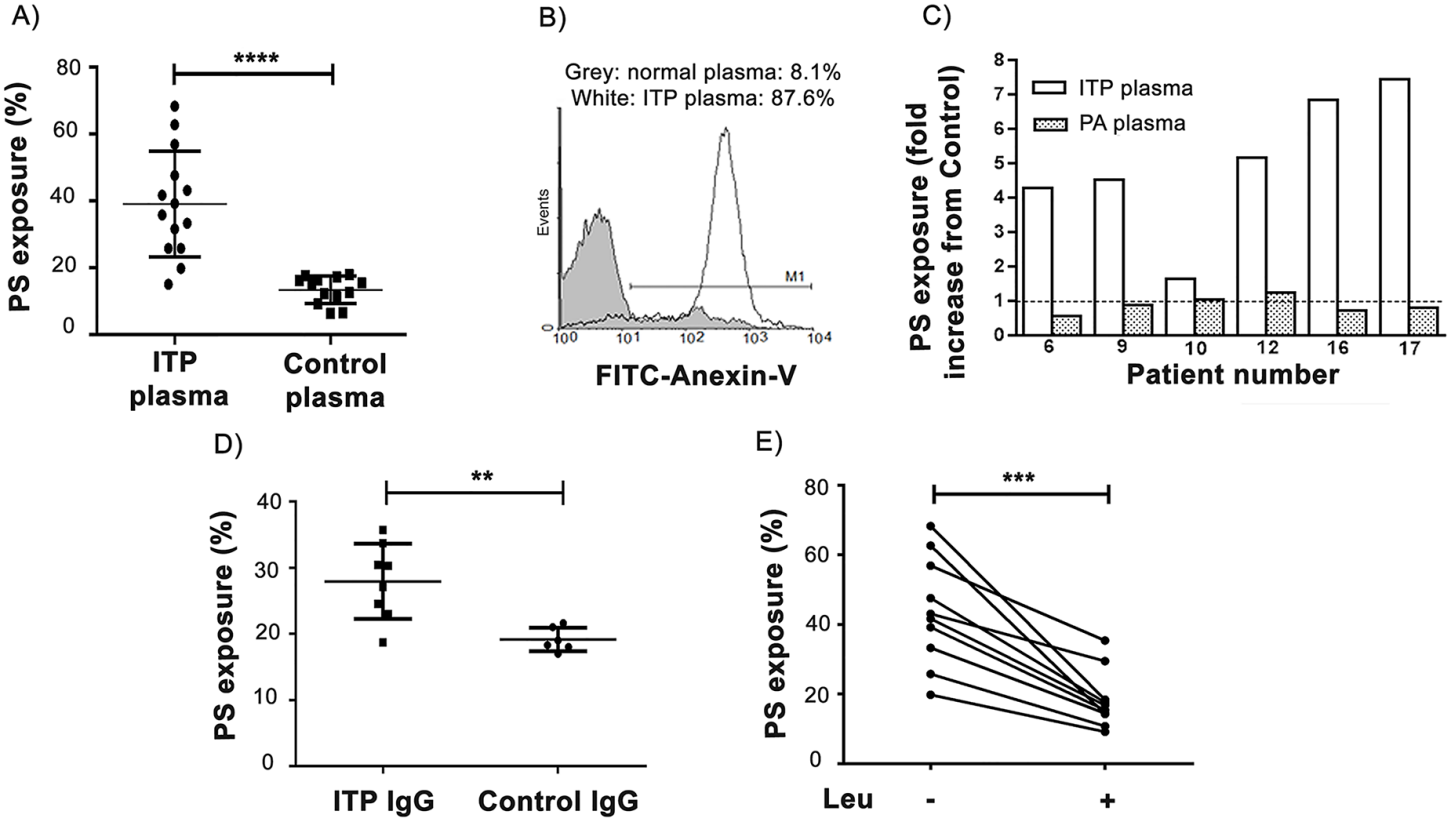
Induction of normal platelet apoptosis by ITP samples in the presence of normal autologous CD3^+^ lymphocytes. Unstimulated normal platelets and autologous CD3^+^ lymphocytes were separately purified and incubated either in ITP or control samples during 1 hour. Then, samples were washed, incubated with FITC-Anexin-V and analysed by flow cytometry within 1 hour of processed to detect PS exposure. A) Percentage of PS expression on normal platelets incubated with ITP plasma (n = 14) and control plasma (n = 13) (Mann Whitney test, ****p<0.0001) in the presence of autologous CD3^+^ lymphocytes. B) A representative example showing percentage of FITC-Anexin-V staining on platelets incubated with ITP plasma and normal plasma is shown. C) Comparison between fold increase in PS exposure on normal platelets incubated with individual ITP recalcified plasma (ITP plasma) and its corresponding platelet-adsorbed plasma (PA plasma), both in the presence of autologous normal CD3+ lymphocytes (Paired t test, p<0.01). Dotted line represents PS exposure of control samples. D) Percentage of PS expression on normal platelets incubated with autologous CD3^+^ lymphocytes in the presence of purified IgG from ITP plasma (n = 8) and control plasma (n = 6) (Mann Whitney test, **p<0.01) E) PS exposure induced by ITP plasma samples in the presence (+) or absence (-) of 20 μmol/L leupeptin (n = 10, paired t test ***p<0.001).

After the finding that ITP plasma had a subtle pro-apoptotic effect on normal platelets in terms of loss of mitochondrial membrane potential, we tested the ability of ITP plasma to further induce PS externalization in normal platelets in the presence of autologous normal CD3^+^ lymphocytes. To this end, ITP plasma samples from 14 patients (four patients with anti-GPIIb-IIIa auto-antibodies, one with anti-GPIIb-IIIa and anti-GPIa-IIa auto-antibodies, one with anti-GPIb-IX auto-antibodies and 8 with no detectable auto-antibodies) along with plasma from 13 healthy controls were used. Incubation of normal CD3^+^ lymphocytes with autologous platelets in the presence of ITP samples induced an increase in platelet PS exposure compared to cells incubated with normal plasma (Fig 4A). A representative example is shown in Fig 4B. Platelet granule secretion, as assessed by surface P-selectin expression, in these conditions was similar in samples incubated with ITP and control plasma (data not shown) ruling out a possible activating effect of ITP samples.

To investigate whether auto-antibodies that could be present in ITP plasma samples could be responsible for the induction of apoptosis in normal platelets, incubation of these plasmas with normal platelets was carried out to eliminate auto-antibodies and, then, the platelet-adsorbed (PA) plasma obtained was tested in our system (normal platelets + autologous normal CD3^+^ lymphocytes). In contrast to the original ITP plasma samples, the corresponding platelet-adsorbed ITP samples were unable to increase PS expression in normal platelets (Fig 4C). Moreover, increased PS exposure was evidenced when purified IgG from ITP samples was used instead of the corresponding whole plasmas (Fig 4D).

Taken together, these results suggest that ADCC is the responsible for platelet damage. To confirm this hypothesis, we added leupeptin, a cathepsin B inhibitor [18], to this system to inhibit the action of one of the main enzymes involved in ADCC. In these conditions, PS exposure was statistically lower than that found in the absence of the inhibitor (Fig 4E).

### Markers of apoptosis in ITP platelets and correlation with biochemical and clinical data

#### Platelet count

All apoptotic parameters evaluated in ITP platelets in resting conditions inversely correlated with platelet count (PS expression, Spearman correlation, R = 0.448, p = 0.0131; loss of ΔΨm, Spearman correlation, R = 0.523, p = 0.0013; aCasp3, Pearson correlation, R = 0.599, p = 0.0394).

#### Presence and specificity of auto-antibodies

**Fig 5:**
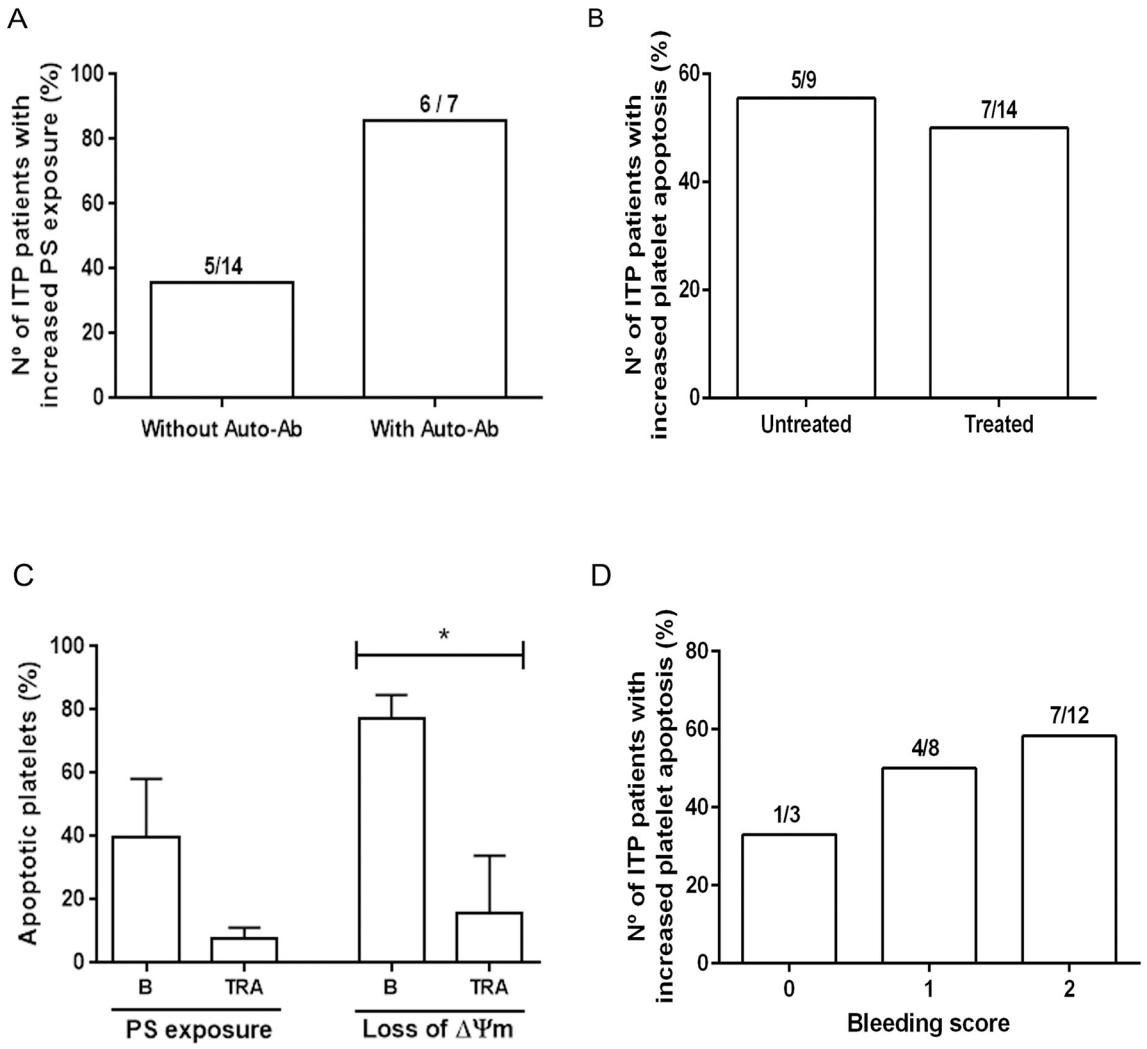
Apoptosis in ITP platelets according to clinical and laboratory data. A) ITP patients were grouped according to the presence/absence of auto-antibodies and increased or normal PS exposure on autologous platelets. The incidence of platelet apoptosis tended to be higher in patients with auto-antibodies than in those with no-detectable auto-antibodies (Fisher exact test, p = 0.063, OR: 10.8, 95% CI = 0.99–117). B) The incidence of platelet apoptosis was similar in patients without treatment and under any kind of treatment (Fisher exact test, p = 1.00, OR: 1.429, 95% CI = 0.271–7.521). C) PS exposure and loss of ΔΨm before (B) and during eltrombopag treatment (TRA). Data represents mean+SD from three ITP patients (Paired t test, PS, p = NS; loss of ΔΨm *p<0.05). D) ITP patients were grouped according to their Bleeding scale (ITP Bleeding Scale-IBLS) and the number of ITP patients with increased platelet apoptosis was plotted in each group (Chi squared test, p = 0.732).

There was a trend for higher incidence of platelet apoptosis in ITP patients in whom auto-antibodies were detected (86%) than in those where auto-antibodies could not be found (36%), as assessed by PS exposure (Fisher exact test, p = 0.063, OR: 10.8, 95% CI = 0.99–117). All patients carrying anti-GPIIb-IIIa auto-antibodies (n = 5) and the one with anti-GPIb auto-antibodies had increased platelet PS. Interestingly, the only patient bearing detectable auto-antibodies without increased platelet PS was the one who had isolated anti-GPIa-IIa auto-antibodies (Fig 5A).

#### Platelet apoptosis and treatment

Platelet apoptosis parameters were similar when comparing untreated patients and patients under any kind of treatment (Fig 5B). However, it must be considered that patients enrolled in this study had moderate to severe thrombocytopenia (range 6–85 x 10^9^/L) regardless of treatment condition. In fact, platelet counts were within the same range in both groups (35±16 x 10^9^/L in the untreated group and 41±22 x 10^9^/L in the group receiving any kind of treatment). During the course of this study, PS exposure and ΔΨm were evaluated in platelets from three ITP patients who received eltrombopag, a nonpeptidyl agonist of the Thrombopoietin receptor (TRA). Both markers decreased during eltrombopag administration in treated patients when platelet count increased (one had a complete response and two other had minor increases in platelet counts) although statistical difference was only observed for ΔΨm (Fig 5C).

#### Bleeding and thrombotic events

Taking into account that PS exposure on platelet membrane could be associated with an increased procoagulant activity, we tried to correlate platelet apoptosis in ITP patients with thrombotic and bleeding complications. None of the patients in this cohort experienced thrombotic events, precluding analysis of a potential association between the procoagulant capacity of platelets and thrombosis. Then, based on the premise that increased platelet procoagulant activity could protect from bleeding, we searched for a possible association between platelet apoptosis and the bleeding score. However, no relationship was found between both parameters in our cohort of ITP (Fig 5D).

#### Reticulated platelets

Patients with ITP had increased percentage of reticulated platelets (13.6%, 3.0–32.3%) compared to normal controls (4.8%, 0.6–13.2%), (Wilcoxon rank sum test, p<0.0001). Levels of reticulated platelet inversely correlated with platelet apoptosis measured in terms of PS exposure (Spearman corelation, r = -0.6295, p = 0.0039) probably due to the fact that young platelets are more resistent to apoptosis.

## Discussion

In the present study, we demonstrate an apoptotic behavior of platelets from ITP patients, as assessed by disruption of mitochondrial membrane potential, increased PS exposure and caspase 3 activity. In agreement with data from Alvarez and col [22], PS externalization was not due to platelet activation because PAC-1 binding and P-selectin expression were normal in ITP platelets in resting conditions and were not increased after agonist stimulation, while agonist-induced decrease of GPIb was also normal. Our finding that apoptotic features, such as PS exposure and altered mitochondrial membrane potential, are linked to caspase 3 activation, which represents a central step in the apoptotic cascade, together with the absence of platelet activation indicate that, as previously shown for pediatric acute ITP [14], platelet apoptosis is a prominent feature of chronic ITP in adult patients.

It is largely recognized that accelerated clearance of auto-antibody-bound-platelets by the reticulo-endothelial system is responsible for thrombocytopenia in ITP. Although this mechanism is still central for elimination of circulating ITP platelets, other pathologenic mechanisms were described, including a cytotoxic T-cell lytic effect [9] and impaired platelet production [3]. Our results show an inverse relationship between loss of ΔΨm, increase in aCasp3 and PS exposure and platelet counts, which highlights the relevance of platelet apoptosis in the development of thrombocytopenia in this disorder and further reinforces the fact that heterogeneous mechanisms contribute to ITP pathogenesis.

The higher incidence of apoptotic platelets in ITP patients carrying auto-antibodies against the major platelet glycoproteins suggests a causal role for these antibodies in triggering platelet apoptosis. Platelet apoptosis was evident in all five ITP patients with anti-GPIIb-IIIa auto-antibodies and the one with anti-GPIb auto-antibodies. The possible link between anti-platelet antibodies and platelet apoptosis has been previously suggested by Leytin et al [13], who described that injection of anti-GPIIb antibodies in a murine ITP model triggers thrombocytopenia that is associated with platelet apoptosis.

In order to assess the contribution of anti-platelet antibodies to platelet apoptosis, in the present study we developed a two-step *in vitro* system to evaluate whether ITP patient plasma could trigger apoptosis of normal platelets. First, normal platelets were challenged with ITP plasma and, next, normal platelets were co-incubated with autologous normal CD3^+^ lymphocytes in the presence of ITP plasma. The first approach showed an increased disruption of mitochondrial membrane potential, as an early apoptotic marker, while PS exposure was not significantly altered. These results point to a soluble factor as responsible for triggering mitochondrial damage. Candidate factors leading to this phenomenon include anti-platelet antibodies and molecules such as TNFα, which could induce apoptosis through the extrinsic pathway. However, TNFα levels were found decreased in ITP [23]. Besides, the responsibility of this non-specific apoptotic factor seems unlikely, since it could induce apoptosis in other hematopoietic cells, leading to cytopenias other that thrombocytopenia, which is not the case in ITP. In our working conditions, complement-mediated cytotoxicity after auto-antibody binding also seems unlikely, since complement was undetectable in recalcified plasma (data not shown).

In the second approach, ITP plasma was shown to trigger full-blown PS exposure in normal platelets when co-incubated with autologous normal CD3^+^ lymphocytes. In this condition, platelet PS exposure reached similar levels to those observed in circulating platelets from ITP patients. To investigate if anti-platelet antibodies represent the plasmatic factor responsible for this effect, we tested the ability of immune-adsorbed plasma as well as purified IgG from ITP to induce PS exposure in normal platelets in this system. Immunodepletion was able to abolish the increase in platelet PS exposure caused by the corresponding original unmodified plasma, while purified IgG reproduced the apoptotic effect. These results point to anti-platelet antibodies as responsible for the observed phenotype. The fact that some of these ITP plasmas were found negative in the assay used for platelet antibody detection, strongly suggest the presence of undetected auto-antibodies in these samples and reflect current limitations for platelet auto-antibody detection in ITP [24].

Previous reports described the contribution of cellular immune mechanisms to ITP platelet destruction. In this regard, Zhao et al [10] demonstrated that ITP CD8^+^ lymphocytes induce platelet apoptosis, involving the participation of HLA type I on CD8^+^ lymphocytes and an abnormal platelet self-antigen presentation. In addition, Olsson et al [9] reported that several cytotoxic genes are overexpressed in ITP CD3^+^ cells and that CD3^+^CD8^+^ cells have a lytic effect on platelets whereas CD3^-^CD56^+^ natural killer cells do not play such a role. In our system, anti-platelet antibodies seem to be essential for reproducing the apoptotic behavior of ITP platelets. The fact that plasma-induced platelet apoptosis was maximal in the presence of normal CD3^+^ cells points to antibody-dependent cell cytotoxicity as the mechanism underlying this phenomenon, suggesting that, besides the already reported direct CD8^+^ cytotoxic effect, T-cells may mediate ITP platelet destruction by an antibody-dependent mechanism. Among CD3^+^ lymphocytes, the γδ fraction could account for this activity [25]. Studies are in progress to identify the lymphocyte subset responsible for this effect.

Although less frequently than in ITP patients carrying auto-antibodies, 36% of the patients in whom platelet auto-antibodies could not be detected, also had increased platelet apoptosis. A possible explanation for this phenomenon could be the presence of auto-antibodies other than those detected by our method or below the threshold of detection of the assay, at least in some samples. The fact that immunodepletion was able to revert the pro-apoptotic effect of plasma and that purified IgG was able to reproduce it, even in those samples without detectable antibodies, further reinforces this possibility.

Interestingly, the patient with anti-GPIa-IIa did not show platelet apoptosis. This type of auto-antibodies seems to behave differently than anti-GPIIb-IIIa and GPIb. In a previous study, we demonstrated that ITP plasma carrying anti-GPIIb-IIIa and GPIb antibodies inhibit proplatelet formation from normal cord blood-derived megakaryocytes, while anti-GPIa-IIa auto-antibodies did not. Moreover, collagen was unable to induce the expected inhibition of proplatelet formation in normal mature megakaryocytes exposed to anti-GPIa-IIa-bearing ITP plasma [3], suggesting an aberrant intramedullary proplatelet generation. Therefore, thrombocytopenia in these patients seems not be due to platelet apoptosis but to other mechanisms including abnormalities in platelet production.

Platelet abnormalities decreased during treatment with eltrombopag in three ITP patients in this study. In constrast to our findings, Álvarez Román and col [22] showed persistent elevated PS expression in patients treated with eltrombopag or romiplostim. Interestingly, Beau Mitchell and col. [26] recently described ameliorated sensitivity to platelet apoptosis in a Bcl-xL inhibitor assay during the first week of treatment with thrombopoietin receptor agonists, together with activation of the pro-survival Akt signaling pathway, although this improvement was not sustained on the second week of treatment. Further work regarding the potential anti-apoptotic effect of thrombopoietin receptor agonists on platelets seems warranted.

In conclusion, this study confirms the relevance of platelet apoptosis in ITP and highlights the role of auto-antibodies in its development. These findings underscore the fact that diverse underlying mechanisms contribute to platelet destruction in ITP and emphasize that both humoral and cellular immune mechanisms, as well as the interplay between them, are involved in ITP pathogenesis.
